# Preimplantation genetic testing and prenatal diagnosis of TANGO2 deficiency disorder with biallelic pathogenic variants using single-nucleotide polymorphism-based haplotyping and gap-polymerase chain reaction

**DOI:** 10.3389/fgene.2026.1832329

**Published:** 2026-08-04

**Authors:** Wanna Ke, Xiaojun Wen, Zhanhui Ou, Xiaowu Fang, Junye Huo, Xiufeng Lin, Xubin Zhang, Zhiming Li

**Affiliations:** Reproductive Medicine Center, Boai Hospital of Zhongshan, Zhongshan, Guangdong, China

**Keywords:** breakpoints, gap-PCR, preimplantation genetic testing, prenatal diagnosis, SNP-based haplotype, TANGO2 deficiency disorder

## Abstract

**Introduction:**

TANGO2 deficiency disorder (TDD), characterized by developmental delays, intellectual disability, gait incoordination, speech difficulties, seizures, and hypothyroidism, is a rare genetic disease caused by biallelic pathogenic variants of the *TANGO2* gene (NM_152906.7).

**Methods:**

In this study, we aimed to present a comprehensive approach for the management of TDD through preimplantation genetic diagnosis and subsequent prenatal diagnosis using single-nucleotide polymorphism (SNP)-based haplotyping and gap-polymerase chain reaction (PCR).

**Results:**

Whole-exome sequencing revealed that the proband was a compound heterozygote with c.672C>A (p.Y224*) and an exon 5-6 deletion (c.266_451del (p.E89Tfs^*^124)). We confirmed these two pathogenic variants using Sanger sequencing and quantitative PCR copy number variation (CNV) analysis, respectively. We successfully constructed an SNP-based haplotype using next-generation sequencing (NGS) in the preclinical phase of preimplantation genetic testing (PGT). In the clinical phase of embryo diagnosis, embryos were subjected to PGT, including NGS-based SNP haplotype linkage and chromosomal CNV analyses. Notably, we successfully identified the exact breakpoints of exon 5-6 deletion (NC_000022.11:g.20053363_20057022delinsAGGT, GRCh38/hg38) using long-range PCR and designed a gap-PCR system based on the location of the breakpoints for subsequent prenatal diagnosis.

**Discussion:**

Taken together, our study provides a method for PGT and subsequent prenatal diagnosis of TDD using SNP-based haplotyping and gap-PCR. The two novel pathogenic variants identified in this study extend the mutational spectrum of TDD.

## Introduction

TANGO2 deficiency (TDD; OMIM 616878) is a rare autosomal recessive genetic disorder caused by biallelic loss-of-function variants in the *TANGO2* gene (NM_152906.7) (Kremer et al., 2016; Lalani et al., 2016). This condition primarily affects the brain, leading to developmental delays, cognitive and language disruptions, intellectual and gait impairments, speech incoherence, epileptic seizures, hypothyroidism, and other nonfatal episodic symptoms. Furthermore, when patients experience physiological stressors, such as fever or fasting, their clinical symptoms can worsen, potentially triggering acute metabolic crises. These crises are often accompanied by ataxia, rhabdomyolysis with elevated creatine phosphokinase, liver transaminases, hypoglycemia, hyperammonemia, elevated troponin and aldolase levels, and cardiac dysfunction, such as cardiomyopathy or arrhythmias, which can result in sudden cardiac arrest (Mingirulli et al., 2020; Bérat et al., 2021; Dines et al., 2019; Miyake et al., 2023; Hoebeke et al., 2021; Powell et al., 2021; Schymick et al., 2022; Miyake et al., 2022; Yılmaz-Gümüş et al., 2023; Miyake et al., 2024; Miyake et al., 1993).

The global prevalence of TDD is estimated to be one in 100,000 individuals, suggesting that over 8,000 individuals worldwide may be affected. To date, more than 100 individuals with biallelic pathogenic *TANGO2* variants have been identified (Miyake et al., 2023; Miyake et al., 1993). There is currently no cure for TDD, and treatment focuses primarily on supportive measures to alleviate symptoms and improve quality of life (Miyake et al., 1993).

Preimplantation genetic testing (PGT) is a key strategy in the field of reproductive medicine that offers couples who are at risk of passing on genetic diseases the opportunity to prevent the transmission of these conditions to their offspring. PGT enables the selection of specific genetic defect-free embryos for implantation created using *in vitro* fertilization (IVF) (Handyside et al., 1992; Carvalho et al., 2020). This preventive approach is particularly crucial for conditions such as TDD, as there are no curative treatments and management is predominantly supportive. By selecting embryos without *TANGO2* mutations, PGT provides an opportunity to prevent disease at the earliest stage, ensuring that only healthy embryos are implanted, thereby reducing the emotional and physical burden on families with genetic diseases.

In this study, we identified two novel *TANGO2* mutations, c.672 C>A (p.Y224*) and exon 5-6 deletion (c.266_451del (p.E89Tfs^*^124)), in a patient with TANGO2 deficiency. Pathogenicity analysis indicated that these mutations are pathogenic. Ultimately, with the help of assisted reproductive technology combined with preimplantation genetic testing for monogenic disorders (PGT-M), the couple was able to have a healthy baby.

## Materials and methods

### Patient history and ethical approval

A 9-year-old female patient was admitted to the hospital multiple times because of delayed development, decreased muscle tone, and recurrent episodes of metabolic encephalomyopathy. Her clinical symptoms are summarized in Table 1. Her first admission to the hospital was at the age of 2 years because of weakness in all four limbs following vomiting and diarrhea. Upon admission, her creatine kinase (CK) level had significantly increased to 242,600 U/L (normal range [NR]: 60–200 U/L), indicating potential muscle damage. After treatment, the patient’s CK levels returned to normal.

**TABLE 1:** Clinical characteristics of the patient.

Category	Clinical feature
Neurodevelopmental	Developmental delay
	Intellectual disability
	Speech difficulties
	Vegetative state (post-cardiac arrest)
Neuromuscular	Decreased muscle tone (initial)
	Increased muscle tone (later)
	Gait incoordination
Metabolic	Recurrent metabolic encephalomyopathy
	Rhabdomyolysis (CK↑ to 242,600 U/L)
	Mild hyperammonemia (45 μmol/L)
	Mild hyperlactatemia (2.43 mmol/L)
Cardiac	Cardiac arrest (requiring CPR)
	Left ventricular dysfunction
	Elevated troponin T (0.131 μg/L)
Endocrine	Hypothyroidism (TSH 11.10 μIU/mL)
	Elevated CK-MB (46.9 IU/L)
Others	Elevated platelet count (416 × 10^9^/L)

CK, creatine kinase; CPR, cardiopulmonary resuscitation; TSH, thyroid-stimulating hormone; CK-MB, creatine kinase-MB isoenzyme.

At 4 years of age, the girl experienced respiratory and cardiac arrest, which required cardiopulmonary resuscitation. Following this event, the patient was readmitted with rapidly progressive symptoms culminating in a comatose state, irregular spontaneous breathing, cool extremities, and echocardiographic findings of abnormal left ventricular wall motion with significantly reduced left ventricular systolic function.

Eight months post-resuscitation, at around the age of 5 years, the girl was readmitted due to developmental delay and a vegetative state. Physical examination revealed fair nutrition, cool extremities without cyanosis, and a capillary refill time of 2 s (NR: ≤2 s). Examination by a specialist revealed that the patient was in a vegetative state. She responded with occasional laughter to tickling but could not follow simple commands. Ocular movements and visual tracking were absent; auditory responses were sluggish. She could slowly ingest liquid foods and use chewing and swallowing movements. Muscle tone in all limbs was increased, and abdominal reflexes were present, with brisk knee and Achilles tendon reflexes.

Positive auxiliary examination revealed a high platelet count (416 × 10^9^/L; NR: 100–300 × 10^9^/L), indicating possible inflammation or infection. Thyroid function tests revealed elevated levels of thyroid-stimulating hormone (11.10 μIU/mL; NR: 0.34–5.91 μIU/mL), suggesting hypothyroidism. The myocardial enzyme profile showed elevated levels of CK (210.2 IU/L; NR: 33–200 IU/L) and CK-MB isoenzyme (46.9 IU/L; NR: 0–33 IU/L), indicating cardiac or muscle injury. During hospitalization, the patient was treated with baclofen to reduce muscle tone and levothyroxine to supplement synthetic thyroid hormone. She received comprehensive rehabilitation training, including magnetic hyperthermia therapy, low-frequency pulsed electrical treatment, hyperbaric oxygen therapy, acupuncture, and swallowing, sensory integration, and movement therapy.

One year after the resuscitation, the patient was readmitted with similar symptoms. Auxiliary examinations revealed elevated levels of cardiac troponin T (0.131 μg/L; NR: 0.000–0.014 μg/L), mildly elevated ammonia (45 μmol/L; NR: 12–35 μmol/L) and lactate (2.43 mmol/L; NR: 0.5–2.2 mMol/L) levels, while other biochemical, liver function, myocardial enzyme profiles, humoral immune indicators, and thyroid function tests were unremarkable.

In summary, the patient was preliminarily diagnosed with post-hypoxic-ischemic encephalopathy, hypothyroidism, visual and auditory pathway abnormalities, and recurrent metabolic encephalomyopathy. The proband and their parents signed a consent form approved by the local ethics committee, and the proband underwent further genetic investigation.

### Trio whole-exome sequencing (trio-WES)

Briefly, genomic DNA was isolated from peripheral blood lymphocytes of the proband and parents using a commercial DNA extraction kit (Cat #13343; Qiagen, Hilden, Germany) according to the manufacturer’s instructions. Exomes were captured using the SeqCap Pure Capture Bead Kit (Roche NimbleGen, Madison, WI, United States) and sequenced on an Illumina NextSeq 500 platform (Illumina, San Diego, CA, United States). Clean reads derived from WES were filtered and aligned to a human reference genome (GRCh38/hg38) using the Burrows–Wheeler Aligner (Li and Durbin, 2009). Single-nucleotide variants (SNVs) and insertions and deletions were identified using a genome analysis toolkit and annotated using SnpEff software. The Human Gene Mutation Database (http://www.hgmd.org), ClinVar (http://www.ncbi.nlm.nih.gov/clinvar), 1000 Genomes Project (http://browser.1000genomes.org), dbSNP (http://www.ncbi.nlm.nih.gov/snp), and ExAC (http://exac.broadinstitute.org/) were used to filter the data. Copy number variations (CNVs) were detected using the ExomeDepth R package (v1.1.6). All putative CNV calls were subsequently visually inspected using the Integrative Genomics Viewer (v2.8.0, Broad Institute) to confirm the deletion breakpoints and exclude false-positive calls due to alignment artifacts. The exon 5-6 deletion was manually confirmed by examining the read-depth reduction across the target exons and by inspecting split-read or discordant-read evidence at the junction boundaries, where available.

According to the American College of Medical Genetics guidelines, all variants can be classified as benign, likely benign, variants of unknown clinical significance, likely pathogenic, or pathogenic (Richards et al., 2015).

Sanger sequencing and quantitative real-time polymerase chain reaction (qPCR) were used to confirm candidate pathogenic SNVs and deletion variants in the proband and the parents, respectively. First, Sanger sequencing primer pairs were generated as a forward primer (5′-AGA​AAC​AAC​CCT​TGC​AGT​TCC-3′) and a reverse primer (5′-TCT​GGA​ATC​CAC​TCA​GGC​GAG-3′) and used to amplify c.672 of the *TANGO2* gene. A forward primer was used as the sequencing primer. The PCR products were sequenced using an ABI3500 DNA sequencer (Applied Biosystems, Foster City, CA, United States). Second, the exon 5-6 deletion in *TANGO2* was confirmed using a gene dosage assay based on qPCR, as previously described (Dong et al., 2023). Primer pairs for amplification of exon 5 were generated as a forward primer (5′-CCC​ACT​TTC​TGA​CCA​CTG​ACG-3′) and reverse primer (5′- GAA​CCC​CAC​TTC​CTG​ATG​AGC-3′). Primer pairs for amplification of exon 6 were generated as a forward primer (5′-ACC​CGT​GAT​GCT​GGA​CAA​GA-3′) and a reverse primer (5′-CGT​CAA​AAC​GAT​AGG​ATC​AGG​C-3′). Primer pairs for amplification of glyceraldehyde 3 phosphate dehydrogenase were generated as a forward primer (5′-GTC​AGT​GGT​GGA​CCT​GAC​CT-3′) and a reverse primer (5′- TCG​CTG​TTG​AAG​TCA​GAG​GA-3′). The fold-change was calculated using the 2^−ΔΔCt^ method.

### Establishment of a gap-PCR system to detect exon 5-6 deletion for PGT-M and prenatal diagnosis

A gap-PCR system was established to analyze the presence of exon 5-6 deletions and differentiate between carrier and normal conditions. Gap-PCR amplification of the exon 5-6 deletion was performed to detect the presence of a deletion. By amplifying exons 5 and 6, it is possible to distinguish between carrier status (heterozygous or homozygous mutation) and normal conditions. The exon 5-6 deletion primer pairs were generated as forward primer F1 (5′-GGA​CAC​ACA​TTG​TCC​CCA​CT-3′) and reverse primer R1 (5′-CTG​TGA​CCT​GCA​ACC​TCA​CT-3′). The amplification of the exon 5 primer pair, F2/R2, and exon 6 primer pair, F3/R3, was consistent with the qPCR. Subsequently, all PCR products were subjected to electrophoresis on a 1.2% agarose gel, and products with the exon 5-6 deletion were further analyzed by Sanger sequencing to identify the deletion breakpoints.

### Oocyte retrieval, intracytoplasmic sperm injection (ICSI), and blastocyst biopsy

Informed consent was obtained from patients before the PGT cycle. Standard IVF techniques were used, and controlled ovarian stimulation was performed using a luteal-phase short-acting long protocol. On the 20th day of the menstrual cycle, the patient was administered a daily 1.0 mg injection of a gonadotropin-releasing hormone agonist (Diphereline; Ipsen, Paris, France). After 14 days of downregulation and confirmation that Luteinizing hormone, follicle-stimulating hormone, estradiol, and progesterone levels were optimal, daily injections of 225 IU of recombinant human follicle-stimulating hormone (GONAL-f; Merck Serono, Geneva, Switzerland) were started, with regular B-ultrasound examinations to monitor follicle development. Once the follicle reached a diameter of 18 mm, an injection of 25 μg of recombinant human chorionic gonadotropin (Ovidrel, Merck Serono, Switzerland) was administered intramuscularly to trigger ovulation. Subsequent B-ultrasound examinations were conducted 34–36 h later to monitor follicle development, and negative-pressure suction egg retrieval was performed under ultrasound guidance. Standard techniques employed in IVF treatment included fertilization, embryo culture, blastocyst biopsy, and blastocyst transfer, as previously described (Wen et al., 2023).

### PGT for aneuploidy (PGT-A) and PGT-M

Whole-genome amplification (WGA) was performed using a ChromSwift kit (Yikon Genomics, Shanghai, China) with multiple annealing and looping-based amplification cycles (MALBAC), according to the manufacturer’s instructions. PGT-A analysis was performed using MALBAC-amplified products that underwent purification, digestion and fragmentation, end repair, adapter ligation, DNA purification, PCR enrichment, purification of the PCR product (library), and CNV sequencing on the DNBSEQ-T7 platform (4 million reads) (BGI, Wuhan, China). Uniquely mapped reads were extracted from alignment reads (BAM files). The entire reference genome was divided into 1 Mb non-overlapping observation windows (bins). Read numbers and GC content were calculated for each bin. GC bias correction was applied for every 1% of GC content. ChromGo software (version 1.8.3; Yikon Genomics) was used to graphically display the GC-corrected relative read numbers of each bin for visualization of CNVs.

The PGT-M single-nucleotide polymorphism (SNP) analysis process was as follows. The WGA-amplified products of embryos, as well as the DNA, were extracted from the peripheral blood of both spouses for SNP detection. The detection process included WGA embryo amplification, purification of the amplification product, family sample DNA extraction, DNA amplification, DNA fragmentation, DNA fragment precipitation, DNA resuspension, hybridization, chip washing, extension and staining, and scanning with an Asian Screening Array gene chip instrument (Illumina), followed by analysis using ChromGO software (Yikon Genomics). Sanger sequencing and the established gap-PCR system were used to determine the genotypes of the embryos. Based on these analyses, euploid embryos without disease-causing mutations or other abnormalities were selected and recommended for clinical transfer.

### Embryo transfer and PGD

The couple received standardized genetic counseling from a board-certified genetic counselor before and throughout the PGT cycle. The possibility of transferring carrier embryos, the implications of incidental CNV findings, and the available alternatives were thoroughly discussed, and written informed consent was obtained for all procedures, including the transfer of carrier embryos if no unaffected euploid embryo was available.

Prior to frozen embryo transfer (FET), the endometrium was prepared using a natural cycle. The timing of ovulation was monitored via B-ultrasound. Five days after ovulation, a normal blastocyst, free of pathogenic mutations and CNVs, was thawed and transferred to the uterus. Clinical pregnancy with one fetus was confirmed when an intrauterine gestational sac and fetal heartbeat were detected through B-ultrasound assessment 30–40 days after FET. To verify the accuracy of PGT results, amniocentesis was performed at 16–24 weeks of gestation. Sanger sequencing and the established gap-PCR system were used to detect pathogenic mutations, and the chromosomal G-banding technique was employed to confirm the results of the karyotype analysis.

## Results

### Identification of biallelic novel pathogenic variants in the TANGO2 gene

**FIGURE 1:**
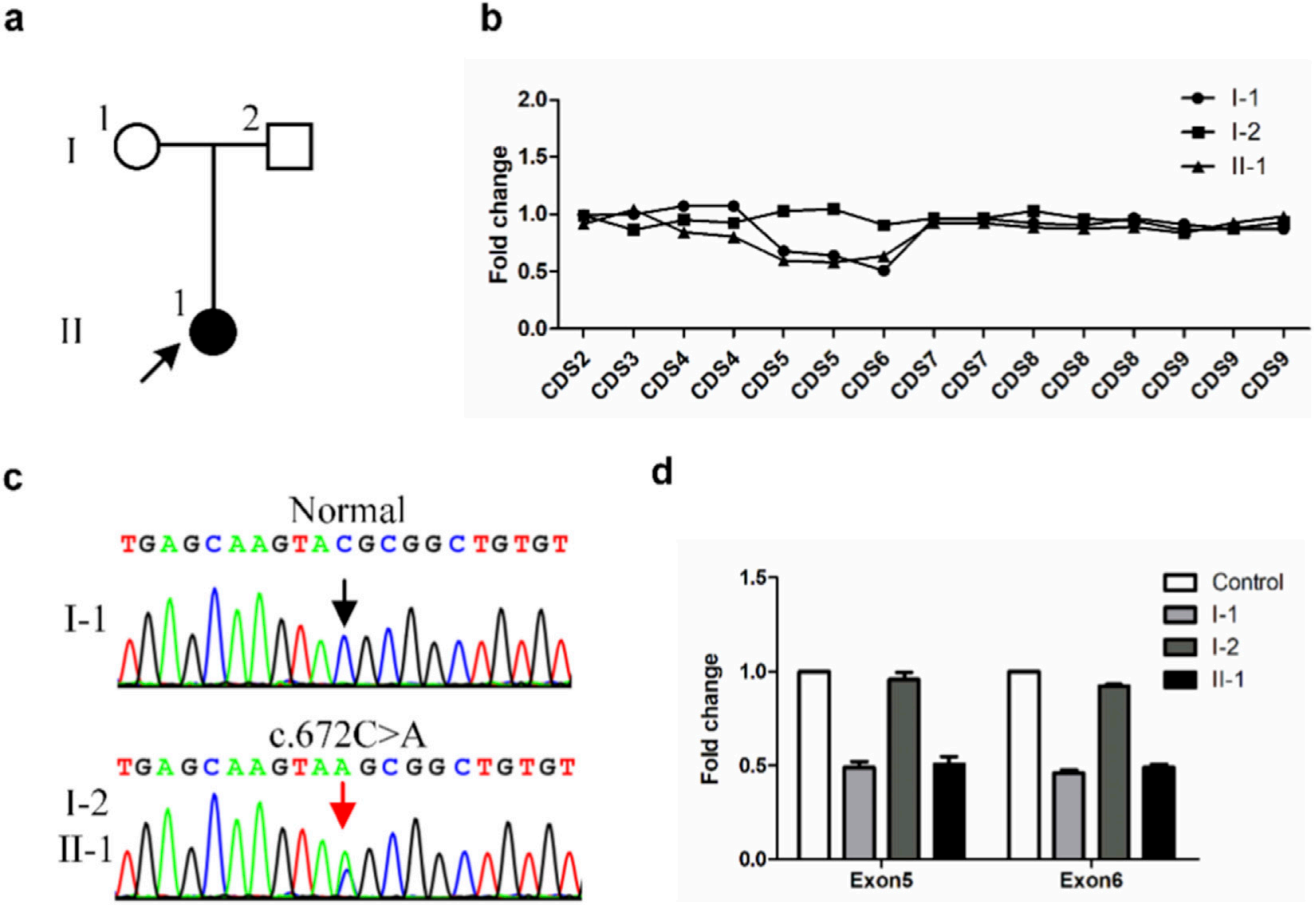
Identification of compound heterozygous mutations in *TANGO2* gene. **(a)** Pedigree of the family. The proband is labeled with arrow. **(b)** Trio whole-exome sequencing (WES) analysis detected copy number variations (CNVs) in the *TANGO2* gene, and the results were normalized by comparing them to normal controls. A value of 1 represents normal, whereas a value of approximately 0.5 indicates a heterozygous deletion. The values of CDS5 and CDS6 in the *TANGO2* gene of the mother (I-1) and proband (II-1) were approximately 0.5, suggesting heterozygous deletions. CDS, coding sequences. **(c)** Sanger sequencing of the nonsense mutation c.672 C>A (p.Y224*) in the *TANGO2* gene. The mutation was observed in the father (I-2) and proband (II-1), but not in the mother (I-1). The black arrow points to the reference position and the red arrow points to the mutant position. **(d)** Quantitative PCR validation of the exon 5 and exon 6 deletion of the *TANGO2* gene. The Y-axis represents the log R ratio, the X-axis represents exon 5 and exon 6, respectively. A value of 1 represents normal, whereas a value of approximately 0.5 indicates a heterozygous deletion.

Trio-WES analysis revealed that the proband (II-1) inherited a heterozygous deletion of exon 5–6 (NM_152906.7 (*TANGO2*):c.266_451del (p.E89Tfs*124)) from her mother (I-1), which was not present in her father (I2) (Figures 1a,b). QPCR validation confirmed the exon 5-6 deletion of the *TANGO2* gene, which aligned with the CNV results from trio-WES analysis (Figure 1d). Furthermore, trio-WES analysis identified the proband (II-1) as heterozygous for the nonsense mutation NM_152906.7 (*TANGO2*):c.672 C>A (p.Y224^*^), which was inherited from the father (I2) and not present in the mother (Figure 1c). Thus, the proband exhibited a compound heterozygous mutation of the *TANGO2* gene involving exon 5-6 deletion and c.672 C>A inherited from her mother and father, respectively. Notably, no mutations were identified in the disease databases (Human Gene Mutation Database, ClinVar) or population databases (dbSNP, ExAC, 1000 Genomes). The pathogenicity of the two variants was assessed according to the American College of Medical Genetics/Association for Molecular Pathology guidelines (Richards et al., 2015). In case of the exon 5-6 deletion the PVS1, deletion is a frameshift mutation, resulting in a change in the reading frame starting from amino acid position 89 and the generation of a premature termination codon at amino acid position 124 downstream. This typically leads to protein truncation or degradation of the mRNA through a nonsense-mediated mRNA decay mechanism, ultimately resulting in loss of function; the PM2_supporting deletion is absent from population databases including gnomAD, 1000 Genomes, and ExAC. Thus, the deletion was classified as likely pathogenic. In case of the c.672C>A (p.Y224*) nonsense variant, the variant PVS1_strong had a premature termination codon present at amino acid 224, which was located in the C-terminal regulatory region (aa 210–276) and predicted to undergo nonsense-mediated mRNA decay or produce a truncated protein; the PM2_supporting variant was absent from all population databases; and the PM3 variant was confirmed to be in trans with the exon 5-6 deletion as the proband inherited c.672C>A from the father and the deletion from the mother. This variant was therefore classified as likely pathogenic.

### Characterization of the deletion breakpoints by gap-PCR and DNA sequence analysis

**FIGURE 2:**
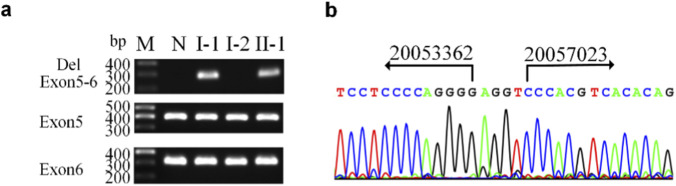
Gap-PCR and Sanger sequencing analysis of the exon 5-6 deletion. **(a)** The gap-PCR system was developed to analyze the presence of exon 5-6 deletion to differentiate between carrier and normal conditions. The electropherograms, arranged from top to bottom, illustrate the gap-PCR amplification of exon 5-6 to determine the presence of a deletion. The middle electropherogram shows the amplification of exon 5, while the bottom electropherogram shows the amplification of exon 6. By combining the three results, the carrier status (heterozygous mutation or homozygous mutations) and normal conditions can be distinguished. In the top electrophoresis, a PCR product of approximately 300 bp was observed in I-1 and II-1 when gap-PCR was performed using primers F1 and R1. No PCR product was observed in I-2 and the control. In the middle, an approximately 400 bp PCR product was amplified in all samples using primers F2 and R2. In the bottom, an approximately 300 bp PCR product was amplified in all samples using primers F3 and R3. M indicates marker and N indicates normal individual. **(b)** Sequencing results of the gap-PCR products. The sequencing results showed that the 5’ breakpoint was located at 20053362, the 3’ breakpoint was located at 20057023, and the deletion size was 3660 bp. The deletion junction inserted AGGT. Therefore, the Human Genome Variation Society designation for the exon 5-6 deletion is (NC_000022.11:g.20053363_20057022delinsAGGT, GRCh38/hg38).

A gap-PCR experiment was conducted to identify the presence of an exon 5-6 deletion to distinguish between carrier and normal statuses. The results indicated that both the proband (II-1) and mother (I-1) were carriers of the *TANGO2* gene exon 5-6 deletion, whereas the father (I-2) did not carry this mutation (Figure 2a). Sanger sequencing revealed that the exon 5-6 deletion was 3660 bp in size, and breakpoints were identified at positions 20053362 and 20057023 on chromosome 22 (GRCh38/hg38) with an insertion of AGGT at the deletion junction (Figure 2b). Therefore, the Human Genome Variation Society designation for the exon 5-6 deletion is (NC_000022.11:g.20053363_20057022delinsAGGT, GRCh38/hg38).

### PGT for the TANGO2 variants

**FIGURE 3:**
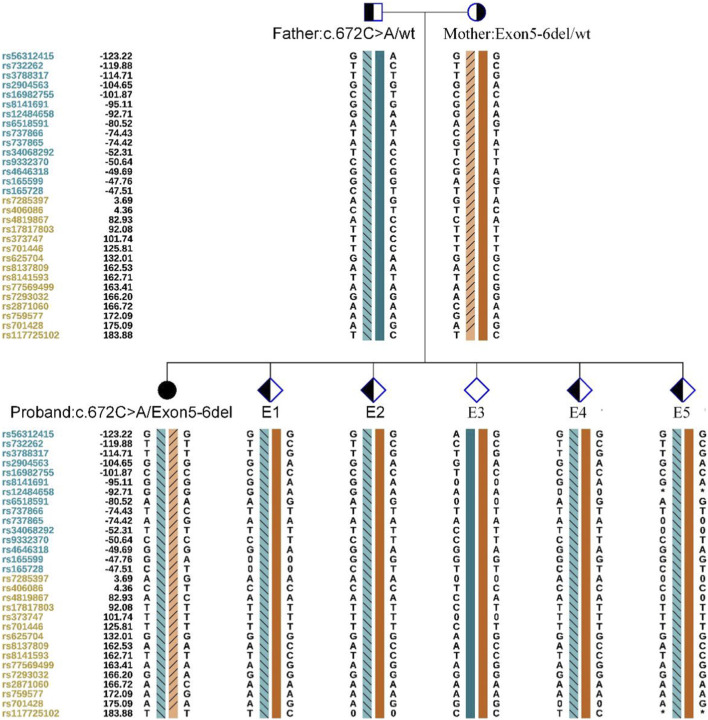
Preimplantation genetic testing for monogenic disorders (PGT-M) for five embryos by haplotype analysis. Green columns containing right slashes represent the pathogenic paternal haplotype with the c.672C>A mutation. Green columns without slashes represent the paternal haplotype without pathogenic mutation. Orange columns containing left slashes represent the pathogenic maternal haplotype with exon 5-6 deletion. Orange columns without slashes represent the maternal haplotype without pathogenic mutation. The leftmost digits represent SNP sites, where blue and orange represent SNP sites upstream or downstream of the chromosome where the gene is located. The black number on the left represents the relative distance (in kb) from the upstream and downstream of the gene. 0 represents the missing SNP information.

**FIGURE 4:**
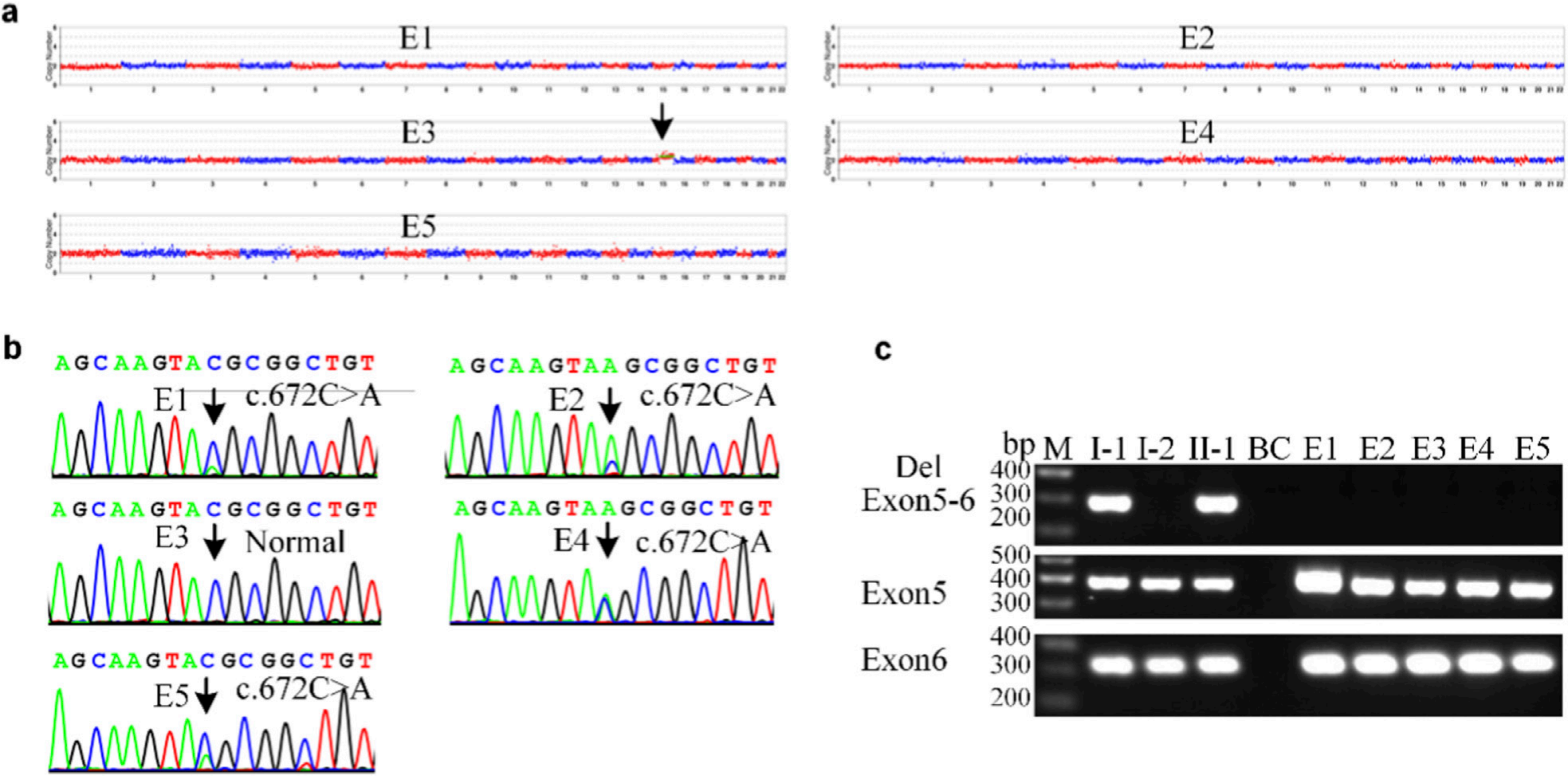
Verification of mutation sites and CNV results in each embryo (E1–E5). **(a)** CNV results of five embryos (E1–E5) using whole-genome amplification–next-generation sequencing (WGA-NGS). A mosaic abnormality was identified in embryo E3, showing a duplication (dup) at 15q21.2q26.3 (∼52.33 Mb, ∼39%), and embryos E1, E2, E4, and E5 were euploid. The black arrow indicates the location of mosaic anomalies. **(b)** Sanger sequencing to verify the mutation in each embryo; the heterozygous mutation (c.672C>A) was observed in embryos E1, E2, E4, and E5 but not in embryo E3. The c.672 position is indicated by the black arrow. **(c)** Gap-PCR system was employed to analyze exons 5 and 6 for both the spouses, the proband, and each embryo (E1–E5). M indicates marker and BC indicates blank control for preimplantation genetic testing (PGT).

The clinical characteristics of the couple treated with PGT are summarized in Table 2. Following ovulation induction, eight eggs were retrieved, all of which reached the metaphase II stage for ICSI. Of these, six eggs were successfully fertilized and developed into normal embryos (two pronuclei). Subsequently, five blastocysts were obtained on day 6 and subjected to biopsy. The biopsied embryos were numbered based on the embryonic morphology score, and trophectoderm cells collected from the biopsy were analyzed for PGT-A and PGT-M. For PGT-M, SNP-based haplotypes were used to identify the embryos carrying pathogenic mutations. The results indicated that embryos E1, E2, E4, and E5 carried the paternal pathogenic mutation (c.672C>A), whereas embryo E3 did not harbor any of the disease-causing mutations (Figure 3). Sanger sequencing confirmed the c.672C>A mutation and exon 5-6 deletion mutation, which was consistent with the SNP-based haplotype analysis results (Figures 4b,c). PGT-A analysis of CNVs revealed mosaicism in dup (Carvalho et al., 2020) (q21.2q26.3) in embryo E3 (∼52.33 Mb, ∼39%), whereas no CNV abnormalities were detected in the other embryos (Figure 4a).

**TABLE 2:** Basic clinical information of the couple and ART embryo development results.

Parameter	Value
Female age (years)	34
Male age (years)	36
Female karyotype	46,XX
Male karyotype	46,XY
Female gene of mutation	NM_152906.7(*TANGO2*):exon5-6del c.266_451del (p.E89Tfs*124)
Male gene of mutation	NM_152906.7(*TANGO2*):c.672C>A(p.Y224*)
Basal sexual hormone FSH (IU/L)	7.61
LH (IU/L)	3.29
T (nmol/L)	0.1
E2 (pg/mL)	44.95
AMH(ng/mL)	3.71
Protocol	
No. of oocytes retrieved	8
No. of mutured oocytes (MII)	8
No. of normal fertilization (2 PN)	6
No. of normal blastocysts	5
No. of blastocysts for biopsy	5

### Clinical outcomes and prenatal diagnosis

**FIGURE 5:**
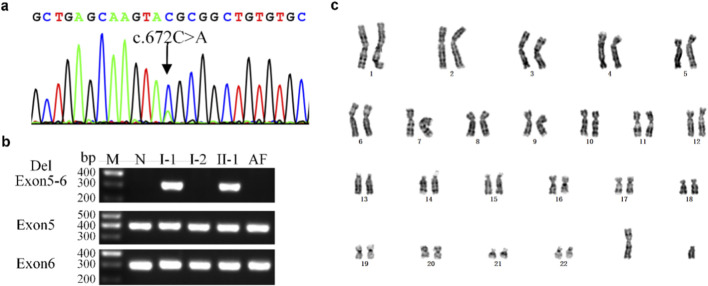
Results of the prenatal diagnosis. **(a)** Results of Sanger sequencing of the amniotic fluid. The c.672 position is indicated by a black arrow. **(b)** Gap-PCR system analysis of peripheral blood and amniotic fluid. M indicates marker, N indicates normal individual, and AF indicates amniotic fluid. **(c)** Results of the chromosomal karyotype analysis.

A comprehensive analysis of the results from PGT (Table 3) revealed that embryos E1, E2, and E4 exhibited morphological scores of 4AA. These embryos also carried the *TANGO2* gene c.672C>A heterozygous mutation and showed no CNV abnormalities. Consequently, E1, E2, and E4 embryos were ranked in transplantation order as first, second, and third, respectively. Conversely, embryo E5 had a slightly lower morphological score of 4AB, carried the *TANGO2* gene c.672C>A heterozygous mutation without any CNV abnormalities, and was placed fourth in the transplantation sequence. Although embryo E3 demonstrated a morphological score of 4AA and lacked a pathogenic mutation in the *TANGO2* gene, the presence of CNV mosaicism [dup (Carvalho et al., 2020) (q21.2q26.3) (∼52.33 Mb, ∼39%)] called for caution during transplantation. Following the transplantation sequence, embryo E1 was thawed and successfully transplanted into the patient’s uterus, resulting in a successful pregnancy. Subsequent amniocentesis at 16 weeks of gestation indicated that the fetus has inherited only the *TANGO2* c.672C>A heterozygous mutation from its father, without the mother’s exon 5-6 deletion mutation (Figures 5a,b). Karyotypic analysis revealed no abnormalities (Figure 5c). These prenatal diagnostic findings aligned with the PGT results. The pregnancy was uneventfully carried to term, and a healthy male infant was delivered at 39 weeks of gestation with normal birth weight (3,250 g) and Apgar scores of 9 and 10 at 1 and 5 min after birth, respectively. The newborn remains healthy with no metabolic or cardiac abnormalities at the 6-month follow-up.

**TABLE 3:** Summary of PGT-M results for five embryos.

Embryo	Score of embryo	The result of CNVs	The result of haplotype analysis	The result of mutation sites verification (sanger sequencing and gap-pcr)	Are the results consistent?
E1	4AA (D6)	46, XN	c.672C>A (paternal)	c.672C>A	yes
E2	4AA (D6)	46, XN	c.672C>A (paternal)	c.672C>A	yes
E3	4AA (D6)	dup (15) (q21.2q26.3) (∼52.33 Mb,∼39%)	normal	normal	yes
E4	4AA (D6)	46, XN	c.672C>A (paternal)	c.672C>A	yes
E5	4AB (D6)	46, XN	c.672C>A (paternal)	c.672C>A	yes

“XN” denotes the sex chromosome complement, where “N” represents either X or Y, consistent with standard cytogenetic nomenclature for embryonic or prenatal samples to avoid disclosure of fetal sex when not clinically indicated.

## Discussion

The *TANGO2* gene is located on chromosome 22q11.21, comprises nine exons and eight introns, and encodes a protein consisting of 276 amino acids. This protein contains three main domains: the N-terminal transmembrane region (Kremer et al., 2016; Lalani et al., 2016; Mingirulli et al., 2020; Bérat et al., 2021; Dines et al., 2019; Miyake et al., 2023; Hoebeke et al., 2021; Powell et al., 2021; Schymick et al., 2022; Miyake et al., 2022; Yılmaz-Gümüş et al., 2023; Miyake et al., 2024; Miyake et al., 1993; Handyside et al., 1992; Carvalho et al., 2020; Li and Durbin, 2009; Richards et al., 2015; Dong et al., 2023; Wen et al., 2023; Milev et al., 2021; Asadi et al., 2023; Sandkuhler et al., 2023; Sacher et al., 2024; Mehranfar et al., 2024; Yan et al., 2023), the central functional region (50–200), and the C-terminal regulatory region (210–276). The exon 5-6 deletion (c.266_451del (p.E89Tfs*124)) is a frameshift mutation that falls entirely within the central functional domain (50–200). Although the specific functions of TDD and the TANGO2 protein have not been fully elucidated, recent studies have suggested that TANGO2 plays a critical role in maintaining the efficiency of endoplasmic reticulum–Golgi membrane transport as well as mitochondrial morphology and function. Disruption of organelle synergy leads to a significant delay in the transport of cargo proteins to the Golgi apparatus (Milev et al., 2021). Previous studies have indicated that TDD impairs lipid metabolism. *In vitro* experiments have demonstrated that vitamin B5 (pantothenic acid) supplementation can rescue TANGO2-dependent cellular transport defects, suggesting that supplementation with multivitamins or vitamin B complex containing vitamins B5 and B3 may benefit patients with TDD (Asadi et al., 2023). A large-scale natural history analysis of TDD revealed that patients supplemented with vitamin B complex or multivitamins had a significantly reduced incidence of metabolic crises and arrhythmias, whereas carnitine and coenzyme Q10 did not exhibit similar effects (Miyake et al., 2023). A natural history study involving 46 patients with TDD found that among children who did not experience metabolic crises, 76% (19/25) were administered multivitamins or vitamin B complex in the long term, whereas none of the children who experienced crises were regularly supplemented (Sandkuhler et al., 2023). Research indicates that TDD is primarily caused by a lipid imbalance. As vitamin B5 serves as a precursor to the lipid activator coenzyme A (CoA), which is a crucial component of lipid synthesis, supplementation with vitamin B5 may alleviate the symptoms associated with TDD and help prevent metabolic crises in patients with TDD (Sacher et al., 2024; Mehranfar et al., 2024).

This study identified a patient with TDD through WES, which revealed a compound heterozygous mutation consisting of a deletion in exons 5-6 of the *TANGO2* gene and a c.672C>A (p.Y224*) mutation inherited from the mother and father, respectively. Owing to the absence of supplementation with multivitamins or vitamin B complex, the proband exhibited severe clinical symptoms, experiencing metabolic crises following viral infections or fasting, which ultimately resulted in respiratory arrest and the current comatose state. The patient displayed a series of clinical manifestations, including developmental delay, intellectual disability, gait incoordination, speech difficulties, seizures, hypothyroidism, rhabdomyolysis, and mild elevations in blood ammonia and lactate levels, all of which are consistent with the known symptoms of TDD (Kremer et al., 2016; Lalani et al., 2016; Bérat et al., 2021; Dines et al., 2019; Miyake et al., 2023; Miyake et al., 1993). The severity and diversity of these symptoms further confirm the critical role of *TANGO2* in the regulation of the nervous system and metabolism.

Among the five biopsied embryos, embryo E3 was the only one free of *TANGO2* mutations; however, it exhibited a mosaic duplication of 15q21.2q26.3 (∼52.33 Mb, ∼39% mosaicism), a finding of uncertain clinical significance. Embryos E1, E2, E4, and E5 were euploid carriers of the paternal c.672C>A mutation without additional CNV abnormalities. After extensive genetic counseling, the couple was fully informed of (i) the asymptomatic carrier status for TDD in the offspring, which poses no health risk; (ii) the uncertain but potentially non-negligible risk associated with the mosaic chromosomal duplication; and (iii) the option to transfer either a carrier embryo or to undergo another IVF cycle. The couple expressed a clear preference for transferring a euploid carrier embryo to avoid the uncertainty of the chromosomal mosaicism. This decision aligns with Chinese experts’ consensus on PGT-M (Yan et al., 2023), which permits the transfer of embryos carrying autosomal recessive mutations as long as the offspring are asymptomatic carriers and the couple has provided informed consent. The transfer of a carrier embryo does not constitute eugenic selection but rather a risk-averse strategy based on the couple’s reproductive autonomy.

There is currently no cure for TDD. The primary treatment measures include daily supplementation with a multivitamin containing all eight B vitamins or at least a vitamin B complex that meets the recommended daily intake to reduce the occurrence of metabolic crises and arrhythmias. Furthermore, providing supportive care enhances quality of life, and offering nutritional support minimizes the risk of cardiac crises. For families with reproductive intentions, once TDD is diagnosed in an affected family member, prenatal diagnosis or PGT can be conducted to prevent the birth of a child with TDD. Previous studies have successfully applied SNP-based haplotyping for PGT-M in various autosomal recessive disorders, including spinal muscular atrophy, β-thalassemia, and cystic fibrosis (Yan et al., 2023). However, for disorders involving complex mutations such as a point mutation combined with a large deletion, integration of direct detection of large deletion mutations (e.g., gap-PCR) with linkage analysis is particularly critical to mitigate the risk of allele dropout and recombination events (Wu et al., 2024). Our approach extends this established framework to TDD. We successfully constructed an SNP-based haplotype using PGT and accurately identified the precise breakpoints of the exon 5-6 deletion. The gap-PCR system designed based on these breakpoint locations can be used to verify the haplotypes of embryos and for subsequent prenatal diagnosis.

In conclusion, this study presents a comprehensive approach for the management of TDD through preimplantation genetic diagnosis and subsequent prenatal diagnosis. The identification of compound heterozygous mutations in the *TANGO2* gene, including a novel nonsense mutation (c.672C>A, p.Y224*) and deletion of exon 5-6, expands the known mutational spectrum of this rare genetic disorder. The absence of these mutations in established disease and population databases highlights their novelty and reinforces the importance of this discovery for genetic counseling and planning for affected families.

## Data Availability

The original contributions presented in the study are publicly available. This data can be found in the CNGB Sequence Archive (CNSA) of China National GeneBank DataBase (CNGBdb), with the accession number CNP0009917.
